# Fish-hook injuries: a risk for fishermen

**DOI:** 10.1186/1746-160X-6-28

**Published:** 2010-12-14

**Authors:** Francesco Inchingolo, Marco Tatullo, Fabio M Abenavoli, Alessio D Inchingolo, Angelo M Inchingolo, Gianna Dipalma

**Affiliations:** 1 Department of Dental Sciences and Surgery, University of Bari, Bari, Italy; 2 Department of Medical Biochemistry, Medical Biology and Physics, University of Bari, Bari, Italy; 3 Department of "Head and Neck Deseases", Hospital "Fatebenefratelli", Rome, Italy; 4 Department of Dental Sciences and Surgery, University of Bari, Bari, Italy; 5 Department of Surgical, Reconstructive and Diagnostic Sciences, University of Milano, Milano, Italy; 6 Department of Dental Sciences and Surgery, University of Bari, Bari, Italy

## Abstract

Fishing is one of the best known and practiced human activities. However, you should remember that, when casting the hook from the riverbank or grasping it to add bait, fishermen run a real risk of injury if the hook punctures the skin.

Briefly we describe a case where a young, 32-year-old fisherman who was reeling the hook back to shore when it hit him in the face and embedded itself in his upper eyelid. Upon examination, the eye was found to be unharmed and the hook was removed through a small incision and the aid of a local anesthetic.

In the light of this case report, we think it a good idea to advise our friends and patients who we know to be fishermen to wear some form of eye protection as a precaution.

## Introduction

Fishing is one of the best known and practiced human activities. Fishing with a rod and hook is probably the most common and popular form, partly because you can fish from the riverbank or seashore, using your own skill to achieve excellent results. Even though there are no particular precautions or warnings for amateur fishermen, it is important to keep in mind some possible complications related to the sport. In particular, you should remember that, when casting the hook from the riverbank or grasping it to add bait, fishermen run a real risk of injury if the hook punctures the skin. The injury may be limited if there are no vital organs involved but can be extremely dangerous if it affects a delicate area, such as an eyelid or the eye itself.

## Case report

**Figure 1:**
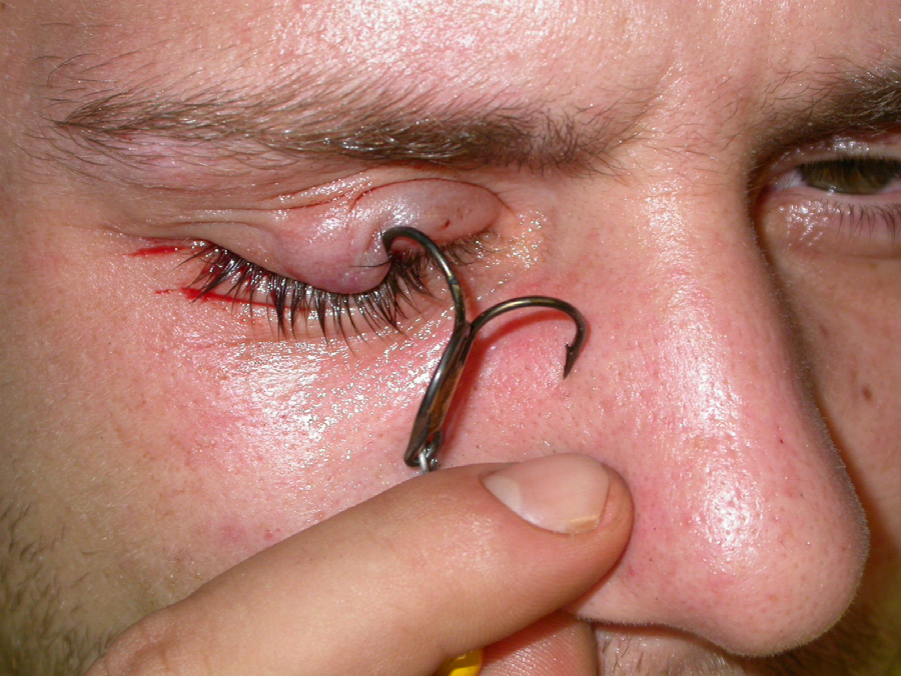
**Photo of the patient right after his arrival in the emergency room with the hook stuck in his upper eyelid**.

**Figure 2:**
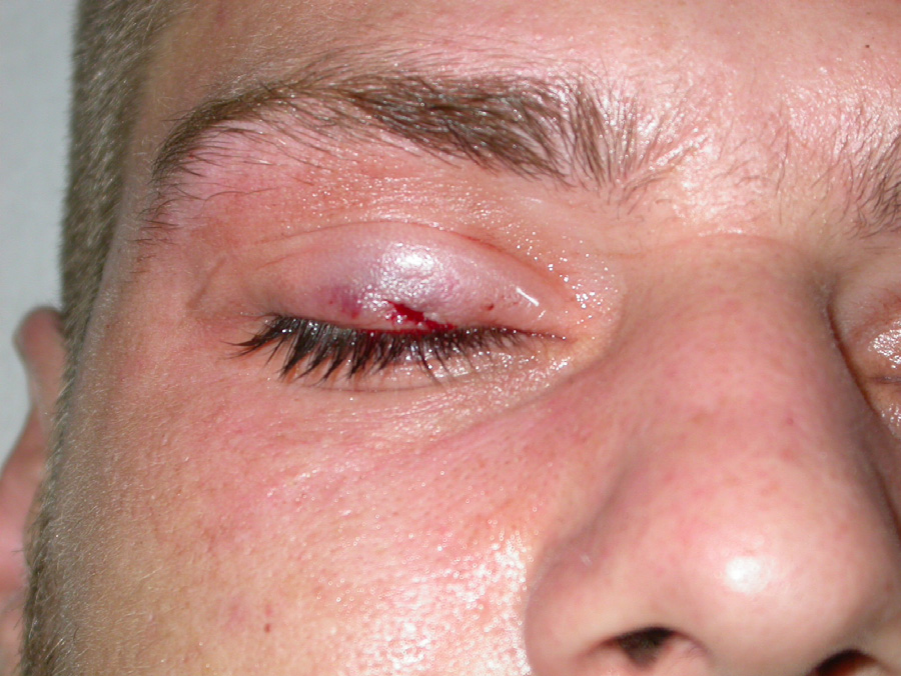
**The hook was removed through a small incision**.

This subject comes to mind because we recently treated a young, 32-year-old fisherman who was reeling the hook back to shore when it hit him in the face and embedded itself in his upper eyelid. The patient was immediately taken to the emergency room (Figure 1). Upon examination, the eye was found to be unharmed and the hook was removed through a small incision and the aid of a local anesthetic (Figure 2). The wound healed normally with no problems for the patient who, being a fishing enthusiast, wanted to go back to the river to pick up where he left off.

## Conclusions

Ocular fishhook injuries can cause severe ocular trauma[1-7]. Aiello et al. reported five cases of penetrating ocular fishhook injuries and showed that with appropriated surgical techniques excellent visual outcome can be achieved in these cases. Appropriate techniques have to be employed to remove the fishhook and avoid major damage to the eyelid anatomy [8]. Penetrating eyelid injury, particularly from fishhooks, is common, with a range of removal techniques available such as retrograde, needle cover, advance and cut, string yank and vertical eyelid-splitting[9]. Considering that medical literature contains many cases of eyelid and eye damage caused by fishing hooks [1-9], we think it a good idea to advise our friends and patients who we know to be fishermen to wear some form of eye protection as a precaution. Fly fishing hooks are very sharp and travel at surprisingly high speeds, for this reason we recommended that all fishermen wear protective eyeglasses similar to those that we use in the operating room to prevent contamination.

## Competing interests

The authors declare that they have no competing interests.

## Authors' contributions

FI, FMA and RC participated in the surgical treatment and in the follow-up examinations. MT drafted the manuscript and revised the literature sources. MM and GD participated in the follow-up examinations. ADI revised the literature sources. AMI managed the data collection and contributed to writing the paper. All authors read and approved the final manuscript.

## Consent statement

Written informed consent was obtained from the patient for publication of this case report and accompanying images. A copy of the written consent is available for review by the Editor-in-Chief of this journal.
